# Real-Time Monitoring of Energy Contributions in Renewable Energy Communities Through an IoT Measurement System

**DOI:** 10.3390/s25051402

**Published:** 2025-02-25

**Authors:** Francesco Bonavolontà, Annalisa Liccardo, Fabio Mottola, Daniela Proto

**Affiliations:** Department of Electrical Engineering and Information Technology, University of Naples Federico II, Via Claudio 21, 80125 Naples, Italy; francesco.bonavolonta@unina.it (F.B.); fabio.mottola@unina.it (F.M.); daniela.proto@unina.it (D.P.)

**Keywords:** renewable energy communities, energy monitoring, energy contributions, smart power meter, IoT measurements

## Abstract

This paper presents an IoT-based monitoring system designed to measure energy exchanges within Renewable Energy Communities. The proposed system utilizes embedded devices functioning as IoT power meters, which communicate via LoRaWAN technology and employ the MQTT protocol. Members of the energy community can monitor energy flows in real time, enabling them to remain constantly informed about potential penalties and adopt behaviors that optimize incentives linked to the self-consumption of generated energy. Moreover, in the case of Renewable Energy Communities, incentive schemes can be adopted which allow exploiting the advantages of using storage units. In this context, it is important to correctly measure the energy terms which can be incentivized. This is not an easy task, especially when dealing with storage units for which the concept of *negative energy input* is used to identify the energy absorbed from the network to be fed back into the network when needed. This paper aims to propose the use of distributed power meters to identify the various energy contributions relevant for incentive calculations, such as negative energy input, produced and withdrawn energy, and self-consumed energy. A case study, involving some resources of a Renewable Energy Community, is presented, evidencing the advantages of the proposal.

## 1. Introduction

Renewable Energy Communities (RECs) offer an innovative model for energy generation and consumption, prioritizing local control, community participation, and sustainability. These communities consist of individuals or organizations collaborating to invest in and manage renewable energy sources, such as solar panels, wind turbines, or biomass systems [1]. RECs are typically rooted in specific geographic areas, enabling members to actively participate in and benefit from local energy initiatives. They focus on sustainability by leveraging renewable energy to reduce carbon emissions and environmental impact. By pooling resources and sharing the benefits of energy generation, RECs often achieve lower costs and greater energy independence. Governance is usually based on democratic principles, allowing decisions to be made collectively by the members. The European Union has strongly supported the development of RECs through the Clean Energy for All Europeans package [2], which ensures they can participate in energy markets alongside larger entities.

The advantages of RECs include the following:

- Enhanced energy efficiency: Localized energy management helps reduce waste and optimize consumption.
- Lower energy costs: Shared investments and collaborative management result in cost savings for participants.
- Improved energy security: Local energy generation reduces reliance on external supplies.
- Strengthened community ties: RECs promote social cohesion and a shared sense of purpose [3].

Providing REC members with insights into energy value offers significant benefits. For example, participants can monitor in real time both the risk of penalties and the potential incentives accrued at the end of the month [4]. Moreover, access to real-time energy flow data encourages behavior that optimizes energy usage to maximize incentives [5,6].

Consider a scenario where the REC informs members about an energy surplus during a specific time period. Instead of feeding this surplus into the grid, members could prioritize self-consumption, such as by scheduling the charging of electric vehicles. This approach fosters both economic and environmental benefits.

For this purpose, the objective of this research is to develop a low-cost distributed REC monitoring system in which smart power meters are to be installed at determined points of the plant in order to monitor energy trends in real time. It is worth specifying that in this application, the real-time specification is intended as soft real-time, i.e., timing constraints are important for performance, but not critical.

The energy quantities to be kept under observation in a REC are different, based on the actual configuration of the REC and the incentive scheme of the self-consumption among members. In particular, depending on the actual location of loads and resources belonging to the REC, the measured energy exchanges need to be differently considered. With reference to the storage units (SUs), the Negative Energy Input (NEI) has been introduced to quantify the portion of energy exchanged by the SU that is useful for remuneration. The Italian Regulatory Authority for Energy, Networks, and Environment (ARERA) has defined the NEI, in Resolution 109/2021/R/eel, as the sum of the electrical energy withdrawn from the network and subsequently fed back into the grid [7].

The contributions of this paper focus on the proposal of distributed power meters capable of identifying the various energy contributions significant for the estimation of the incentive calculations such as the negative energy input and the produced and the withdrawn energy. For this purpose, an Internet of Things (IoT)-based monitoring system is designed to measure the energy exchanges of the REC members. Particular attention has been given to cost limitation. The monitoring system involves the deployment of multiple sensor nodes within the REC. Therefore, cost has been a key criterion in the selection of hardware components, transmission technologies, and software processing.

More specifically, we propose the use of embedded devices functioning as IoT power meters, which communicate via Long Range Wide Area Network (LoRaWAN) technology and employ the Message Queuing Telemetry Transport (MQTT) protocol.

Compared with the conference paper [8], the following extensions have been added: (i) the measurement system has been improved, by leveraging a 0.2% accuracy power meter by STMicroelectronics; (ii) the model of the REC operation strategy has been detailed; (iii) the charging/discharging patterns of the SU have been analytically detailed to highlight the terms needed for the correct evaluation of the shared energy and then those on which masurements are required; and (iv) new results have been included.

The organization of the paper includes, in Section 2, the presentation of the REC under study and the operation strategy for optimizing their resources, as well as the configurations allowed for the SUs together with the required use of the measurement systems according to the allowed schemes for the incentive. The requirements of an IoT monitoring system, properly tailored for REC applications, are detailed in Section 3. In Section 4, the results of a numerical application regarding a measurement example concerning resources in a REC connected to a low voltage (LV) distribution network are discussed. Conclusions are reported in Section 5.

## 2. Measurement of the Energy Exchanges in an REC

To estimate the energy exchanges, it is required that the different energy contributions associated with production, storage, self-consumption, and withdrawal be separated. The location of the resources in the network is also important for effectively quantifying the energy quota subject to the incentive. For this purpose, in this Section, the REC under study is detailed first, and a possible operation strategy for its resources is briefly discussed. Then, the way this strategy can be applied by properly measuring the energy exchange under different configurations of SUs is discussed.

### 2.1. REC Configuration and Operation Strategy

The REC under study includes members characterized by the presence of at least one of the following: a consumption unit (CU); a production system (PS) based on renewable energy sources (RESs), such as photovoltaic (PV) systems; a and storage unit (SU). During the charging stage, the SU is supposed to be able to absorb power from both the PS and the grid. During the discharging stage, the SU is supposed to be able to feed the CU and feed back into the grid. A proper control strategy for the SUs must be considered, to control the energy exchanges among the resources to maximize the shared energy while guaranteeing the correct balancing of the stored energy and providing a specified power to the network.

The SU control strategy is defined in more detail below.

During the charging stage, the energy charged by the SU is as follows:

(1) $$E_{a,t,i}=\eta_{ch}\left(E_{a,PSU,t,i}+E_{a,SU,t,i}\right)$$

where, with reference to the *i*th SU and time *t*; $E_{a,t,i}$ is the total energy charged by the SU; $E_{a,PSU,t,i}$ is the energy absorbed from the PS; and $E_{a,SU,t,i}$ is the energy absorbed from the grid, and $\eta_{ch}$ is the charging efficiency.

During the discharging stage, the energy discharged by the SU into the grid is as follows:

(2) $$E_{p,t,i}=\frac{1}{\eta_{dch}}\left(E_{p,SU,t,i}+E_{p,SUn,t,i}+E_{p,CU,t,i}\right)$$

where, with reference to the *i*th SU and time *t*, $E_{p,t,i}$ is the total energy discharged by the SU; $E_{p,SU,t,i}$ is the energy fed into the network and previously charged from the PS; $E_{p,SUn,t,i}$ is the energy fed into the network and previously withdrawn from the network; $E_{p,CU,t,i}$ is the energy discharged to supply the CU; and $\eta_{dch}$ is the discharging efficiency of the SU.

At each time *t*, for each battery *i*, the total energy stored in the SU, $E_{t,i}$, is given by the following:

(3) $$E_{t,i}=E_{0,i}+\sum_{\tau =1}^{t}\left(E_{a,\tau ,i}-E_{p,\tau ,i}\right)$$

where $E_{0,i}$ is the energy stored in the battery at t=0. A proper constraint must be imposed to limit the stored energy within admissible ranges as follows:

(4) $$E_{min,i}\leq E_{t,i}\leq E_{max,i}$$

where $E_{min,i}$ is the minimum value, which can be imposed by technical limitations, such as the maximum depth of discharge to prolong the battery lifetime, and $E_{max,i}$ the maximum value which can be assumed equal to the SU-rated capacity.

A constraint must also be imposed on the charging and discharging power of the SU, whose values cannot exceed the SU-rated power, as follows:

(5) $$\frac{1}{\eta_{ch}}\frac{E_{t+1,i}-E_{t,i}}{\Delta t}\leq P_{SU,i}$$

(6) $$\eta_{dch}\frac{E_{t,i}-E_{t+1,i}}{\Delta t}\leq P_{SU,i}$$

where $P_{SU,i}$ is the rated power of the *i*th SU and Δt is the length of the control time interval. It is worth noting that the power charged/discharged by the SU is obtained by the maximization of the shared energy. Moreover, the SU power can also be used to provide ancillary services or to perform price arbitrage derived from the optimal scheduling of the REC resources (e.g., [9]) whose details are not reported here since they are out of the scope of this paper.

Regarding the shared energy, this is defined as follows:

(7) $$E_{shared,t}=\min\left\{E_{a,rec,t};E_{f,rec,t}\right\}$$

where $E_{a,rec,t}$ is the energy absorbed by the REC’s members and $E_{f,rec,t}$ is the energy fed into the grid, excluding that previously withdrawn and then released from the SU, both related to the time *t*. In particular, $E_{a,rec,t}$ includes both the energy absorbed to supply the CUs and that used to charge the SU, as follows:

(8) $$E_{a,rec,t}=\sum_{i=1}^{N_{m}}E_{a,CU,t,i}+E_{a,SU,t,i}$$

where $E_{a,CU,t,i}$ is the energy absorbed by the *i*th CU and $E_{a,SU,t,i}$ is the energy absorbed from the grid to charge the the SU, both in reference to the time *t*, and $N_{m}$ is the number of REC members. Regarding the energy fed into the grid, $E_{f,rec,t}$ is given by the following:

(9) $$E_{f,rec,t}=\sum_{i=1}^{N_{m}}E_{p,PS,t,i}+\sum_{i=1}^{N_{m}}E_{p,SU,t,i}$$

where $E_{p,PS,t,i}$ is the energy produced by the PS and fed into the grid related to the time *t*. According to [10], the term $E_{p,SU,t,i}$ is estimated based on the evaluation of the NEI whose algorithm depends on the particular configuration of the CU, SU, and PS of each member *i*, as detailed in the next subsection. As mentioned above, the energy fed back into the grid is related to price arbitrage or other services which the SU can offer to the grid.

The implementation of proper algorithms, which allow for identifying the contributions of the energy exchanged by the CUs, SUs, and PSs through the use of appropriate measurements, is detailed in the next subsection.

### 2.2. Measurement of the Energy Exchanges

In [7], the way SU measurements are considered refers to the “Transmission, Dispatching, Development, and Grid Security Code”, which provides the procedures for the connection, management, planning, development, and maintenance of the Italian national transmission grid. Regarding the NEI, Annex A78 of the Code is the document titled “Algorithms for the measurement of Negative Energy Input” [10]. In this document, four types of configurations are considered as follows:

- Type A, involving only the Production System (PS) and/or the Storage Unit (SU).
- Type B, involving the Consumption Unit (CU) and the Production System (PS).
- Type C, involving the Consumption Unit (CU) and the Storage Unit (SU).
- Type D, involving the Consumption Unit (CU), the Storage Unit (SU) and the Production System (PS).

**Figure 1:**
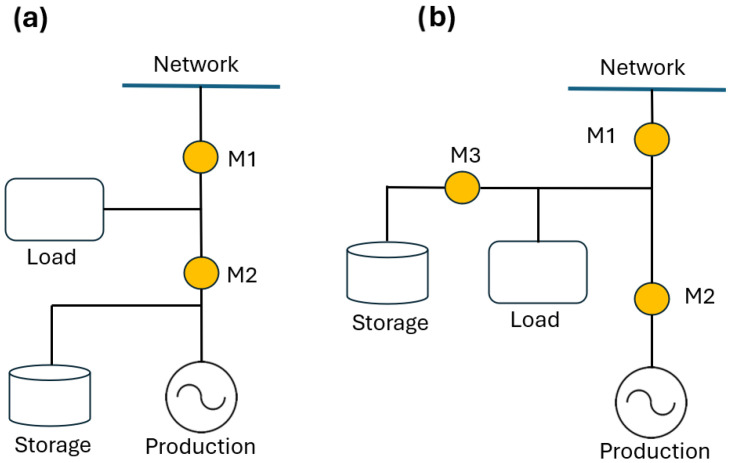
Connection typologies for the SU: (**a**) production-side; (**b**) post-production [11].

This paper focuses on Type D. Based on the relative positioning of the SU and the measurement devices, storage systems can be categorized as follows (Figure 1) [11]:

- Production-side SU: The storage unit is installed within the DC or AC electrical circuit, located between the PS and the device that measures the produced energy. In this configuration, it becomes impossible to differentiate between the energy generated by the production plant and that discharged by the storage unit.
- Post-production SU: The storage unit is associated with a dedicated measurement device and placed between the meter that records the produced energy and the one monitoring the energy exchanged with the network.

**Figure 2:**
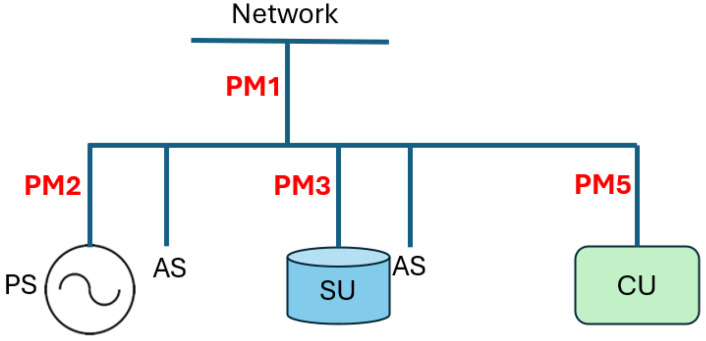
Scheme of the considered system [10].

This research aims to estimate all the energy contributions in the REC. Therefore, in this paper, the focus is on the post-production side configuration, where it is possible to separate the measurement of the energy generated by the PS from that discharged by the SU. Figure 2 shows, therefore, the schematic diagram of the system under study.

Specifically, the scheme considered follows Type D.1.d, as described in [10], where, in addition to the meter that measures the power exchange with the network, three meters are used. The energy consumed by the internal services of both the PS and the SU is not individually measured and is instead grouped with the energy drawn by the CU.

With the placement of the meters, it is possible to determine, at time *t*, the energy production $PM2.EP_{t}$ from the readings provided by power meter PM2. This meter is unidirectional since it is intended to only measure the energy produced.

The power meter PM5 is also unidirectional and records the energy consumption at time *t* for the CU, denoted as $PM5.EC_{t}$. Meters PM1 and PM3 must be bidirectional, as they measure the energy exchange. Specifically, $PM1.EW_{t}$ represents the energy withdrawn from the network, $PM1.EF_{t}$ is the energy fed into the network, $PM3.EA_{t}$ is the energy absorbed by the SU, and $PM3.ER_{t}$ is the energy released by the SU at time *t*.

The most complex measurement is that of the NEI, as the SU can (1) draw energy from both the network and the PS, and (2) supply energy to both the network and the CU.

According to the algorithm in [10], the NEI at time *t* is calculated using the following formula:

(10) $$NEI_{t}=EP_{SA_{t}}+EP_{a_{t}}^{r}$$

The NEI is then given by the sum of two components as follows: (i) $EP_{SA_{t}}$, which is the energy withdrawn by internal services; and (ii) $EP_{a_{t}}^{r}$, which is the energy withdrawn from the grid by the SU to be fed back into the grid subsequently. In this application, the energy withdrawn by internal services is not measured and is therefore associated with the CU’s withdrawal, i.e., $EP_{SA_{t}}=0$.

To quantify the amount of energy re-injected into the grid, the partition coefficient $c_{p}^{m}$ is used. The superscript *m* indicates that this coefficient is calculated on a monthly basis. This is estimated as follows:

(11) $$c_{p}^{m}=\frac{EI_{a_{m}}^{r}}{PM3.ER_{m}}$$

where $PM3.ER_{m}$ is the energy supplied by the SU throughout the previous month. The term $EI_{a_{m}}^{r}$ represents the energy released in the previous month by the SU to be fed into the grid and supply internal services. Its monthly value is calculated by summing, over the entire previous month, the fraction of energy injected into the grid provided by the storage system, as follows:

(12) $$EI_{a_{m}}^{r}=\sum_{t}EI_{a_{t}}^{r}=\sum_{t}PM1.EI_{t}\cdot \frac{PM3.ER_{t}}{PM3.ER_{t}+PM2.EP_{t}}$$

where $PM1.EI_{t}$ is the total energy injected into the grid at time *t*; $PM3.ER_{t}$ is the energy supplied by the storage system at time *t*; and $PM2.EP_{t}$ is the energy supplied by the PS at time *t*.

Thus, the energy withdrawn from the grid and fed back into the grid is estimated as follows:

(13) $$EP_{a_{t}}^{r}=c_{p}^{m}\cdot EP_{a_{t}}$$

where $EP_{a_{t}}$ is the energy withdrawn by the SU from the network at time *t*; it is calculated as a distribution of the energy withdrawn at the exchange point, according to the weight of the storage withdrawal in the total energy withdrawn from the grid. More specifically, this is expressed as follows:

(14) $$EP_{a_{t}}=PM1.EP_{t}\cdot \frac{PM3.EA_{t}}{PM3.EA_{t}+PM5.EC_{t}}$$

## 3. IoT Monitoring System

The monitoring system suitable for the considered configuration is therefore made up of four power meters, arranged at the points of the system shown in Figure 2. The meters must present the metrological characteristics required by the ARERA Resolution; therefore, they must be able to provide an update of the energy values every quarter of an hour.

**Figure 3:**
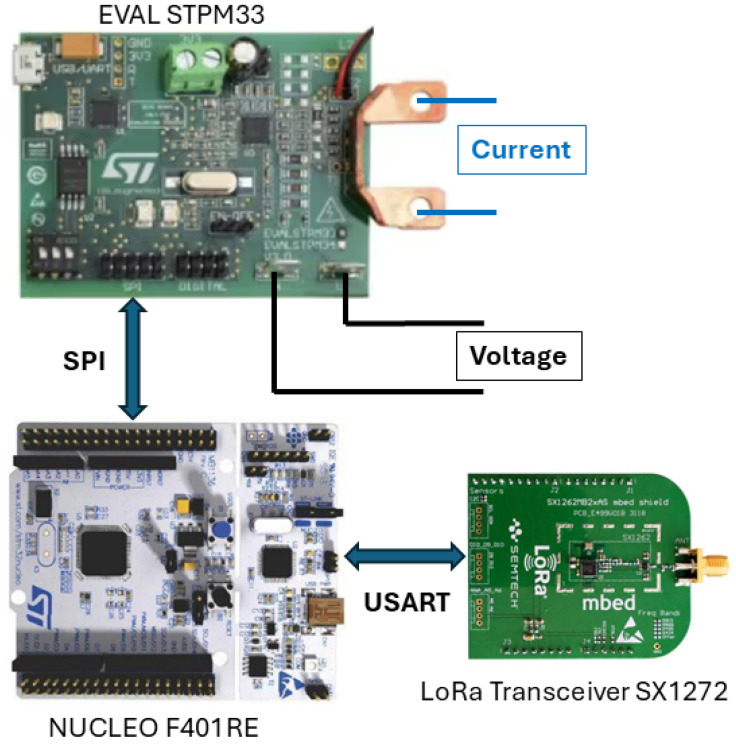
Scheme of the developed smart power sensor.

Regarding the meters presented in [8], the authors chose to take into account a single board power meter in order to enhance the accuracy and reduce the cost. The scheme of the developed smart meter is shown in Figure 3. It is composed of three sections as follows: (1) measurement section; (2) control section; and (3) transmission section.

The measurement section involves the device STPM34 by STMicroelectronics, Geneva, Switzerland which has been used to exploit the evaluation board EVAL STPM33. The STPM34 includes both the analog front end and the digital section to perform high-accuracy power and energy measurements. Its main characteristics are the following:

- Vcc supply range 3.3 V.
- Supply current Icc 4.3 mA.
- Four independent 24-bit second-order sigma-delta Analog to Digital Converters (ADCs).
- Two programmable gain chopper stabilized low-noise and low-offset amplifiers.
- Measurement of the Root Mean Square (RMS) of the voltage and current; active, reactive, and apparent power; active, reactive, and apparent energy; frequency; and voltage-current phase displacement.
- Bandwidth 3.6 kHz at −3 dB.
- Active power accuracy < 0.1% over a 5000:1 dynamic range and <0.5% over a 10,000:1 dynamic range.
- Reactive power accuracy < 0.1% over a 2000:1 dynamic range and <0.5% over a 10,000:1 dynamic range.

The sampling rate adopted by the power meter device is 125 kHz. Every 16 samples, all the measurement data are updated in the respective registers, obtaining an update rate of 7.81 kHz.

The evaluation board provides the transducers to obtain the voltage and current signals. In particular, the voltage is measured by means of a resistive voltage divider (ratio of 1724:1), and the current is measured through a shunt resistor, whose resistance is equal to 0.0003 Ω. Thus, the voltage- and current-rated values are set equal to 300 V and 5 A, respectively. The current, however, can reach the maximum value of 100 A. According to the device clock frequency, signals are sampled at the rate of 7.8125 kHz; as soon as a new sample is gathered, the measures are updated.

The measures can be read from the data registers of the device STPM34 through Serial Peripheral Interface (SPI) communication. To this end, the control section has been realized with a board Nucleo F401RE by STMicroelectronics [12,13] that includes a 32-bit ARM Cortex STM32F401RE, which operates at a frequency of 84 MHz. Due to to its excellent performance relative to its low cost, this board is widely used in IoT monitoring systems The microcontroller accomplishes the following three tasks:

- Configuring the power meter (algorithm for apparent power estimation, gain of the amplifiers in the analog front end, and insertion of filters).
- Reading the measures from the meter register through SPI communication every fifteen minutes. The microcontroller autonomously wakes up every fifteen minutes due to the embedded Real-Time Clock (RTC) [14,15].
- Forwarding the measures to the transmission unit through USART (Universal Synchronous/Asynchronous Receiver/Transmitter) communication.

Finally, a NUCLEO-SX1272D board by ST Microelectronics, which integrates a LoRa SX1272 transceiver has been adopted as the transmission unit of each power meter. Considering the required data transmission rate and the limited volume of data to be sent, LoRaWAN technology appears to be the most appropriate communication solution. One of the key advantages of Long Range (LoRa) transmission lies in the high sensitivity of its receivers, enabling them to accurately demodulate signals with low received signal strength indicator (RSSI) values, even when affected by significant noise. This characteristic ensures that LoRa transmission remains robust and dependable, even over long distances or in scenarios where obstacles interfere with the Line of Sight (LoS) [16,17].

Additionally, compared to power line communication (PLC), a commonly used technology in electrical grids, LoRa offers simpler installation, a benefit shared by other wireless technologies [18,19].

**Figure 4:**
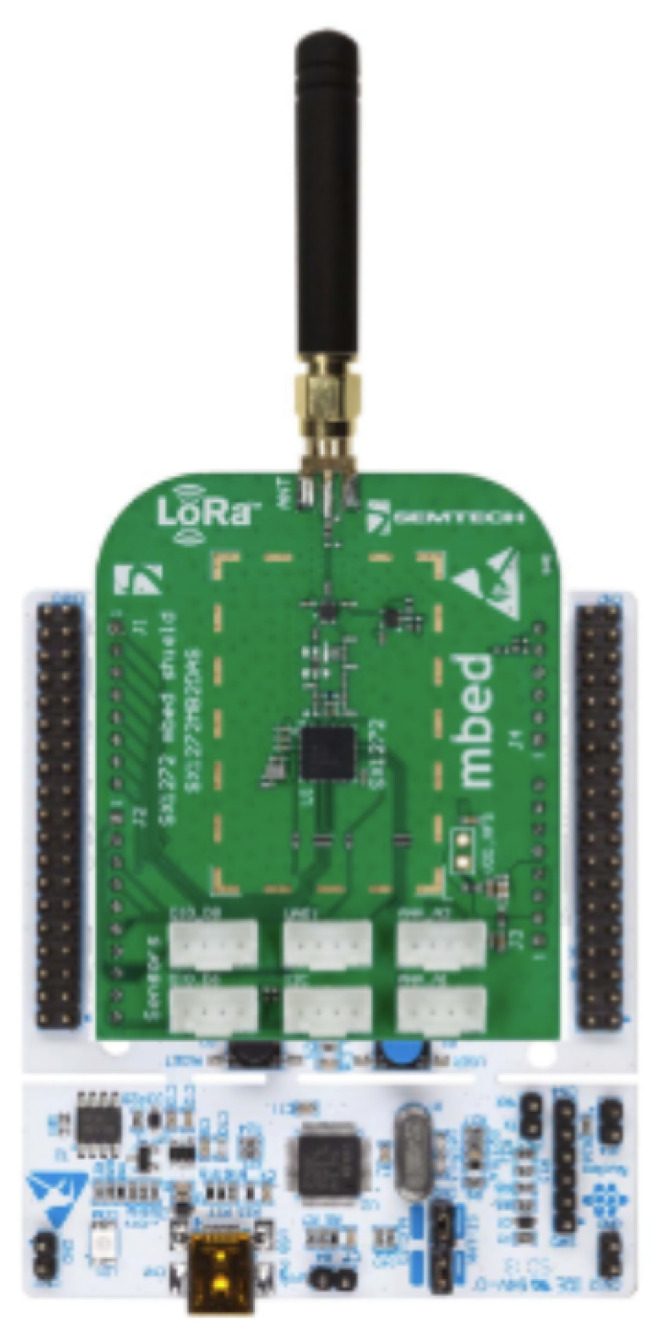
Lora stack realized with a Nucleo board and LoRa transceiver.

Using Arduino-compatible connectors, the extension board is directly attached to the NUCLEO F401RE demo board, creating a hardware stack as illustrated in Figure 4.

The boards interact with a gateway MTCAP-868-001A by MultiTech Systems Inc., Mounds View, MN, USA, which forwards the received messages based on the selected protocol. Regarding the communication protocol, MQTT was chosen primarily for its capability to manage the flow of information from one device to multiple recipients [20,21]. In conventional network communication, which operates on a request/response model, clients and servers exchange data directly as follows: clients request resources or data from the server, which processes the request and responds. Conversely, MQTT adopts a publish/subscribe model, allowing messages to be exchanged through a “message broker”. Instead of directing messages to specific recipients, senders publish messages to a “topic” managed by the broker. Recipients then subscribe to topics of interest, and the broker ensures that messages are delivered to all subscribers whenever new content is published on a specific topic [22,23].

Recent advancements in IoT and Industrial IoT (IIoT) have increasingly leveraged open source and low-cost technologies to develop scalable and flexible monitoring solutions [24]. This trend is particularly relevant in the field of renewable energy, where the deployment of distributed sensor networks enables real-time data acquisition and improved system efficiency [25]. MQTT, as an open source communication protocol, has been widely adopted in IoT ecosystems due to its lightweight nature and reliability in constrained environments. Several recent studies have explored similar approaches in scientific scenarios related to renewable energy research, highlighting the advantages of cost-effective and open source solutions in monitoring and control applications [26]. In this context, the proposed monitoring system aligns with these developments by integrating low-cost hardware and efficient data transmission mechanisms, ensuring both accessibility and adaptability for real-world implementations in Renewable Energy Communities (RECs).

This system enables a monitoring setup where each power meter publishes its measurements to a dedicated topic. Anyone interested in the data from a particular power meter can subscribe to its topic, and any new data published are automatically shared with all subscribers.

This publish/subscribe model not only supports one-to-many communication but also enhances scalability, as adding a new user simply requires subscribing to an existing topic to start receiving data from the power meter.

Data are stored in a database located on a server that acts both as a historical data repository and a controller that runs an eventual algorithm for optimization strategy.

However, the main advantage of using MQTT is that users can receive measurement data on their own computer or smartphone by subscribing to the corresponding topic. This means that users can decide to save data by implementing their own historical data repository or just visualize current data, through a custom dashboard.

As previously mentioned, client software for such a system can be developed using various technologies. In this case, the authors utilized Node-RED 3.0.2, an open source platform known for its flexibility and user-friendly approach to creating IoT solutions [27,28].

**Figure 5:**
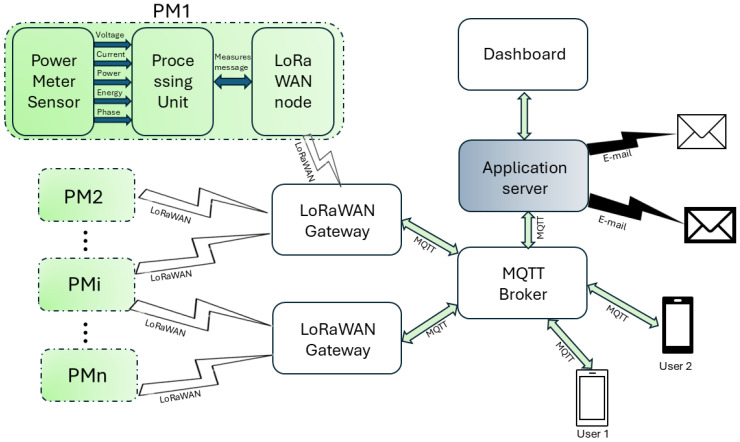
Diagram of measurement data processing stages.

For a better understanding of the implemented architecture, the flowchart of the measurement data is shown in Figure 5, from the power meters (indicated as PM) to the end user.

Each power meter transmits measurement data to the Gateway using LoRaWAN technology. The Gateway then forwards the messages to the broker via an Ethernet connection (although 4G or 5G are also suitable for this purpose) in the form of an MQTT message, which is published under a specific topic; each power meter is associated with a dedicated topic.

The broker, according to the MQTT protocol, forwards the message to all users who have subscribed to the topic. Among them, the authors have realized an Application server, running a Node-RED software, v 3.0.2 which performs the following functions: (1) storing the measurement data from all power meters in a database; and (2) creating the general dashboard, which all members of the REC can view by accessing a web page.

This does not exclude the possibility for each user to develop their own data visualization interface on their preferred device.

## 4. Measurement Example in a LV Distribution Network

**Figure 6:**
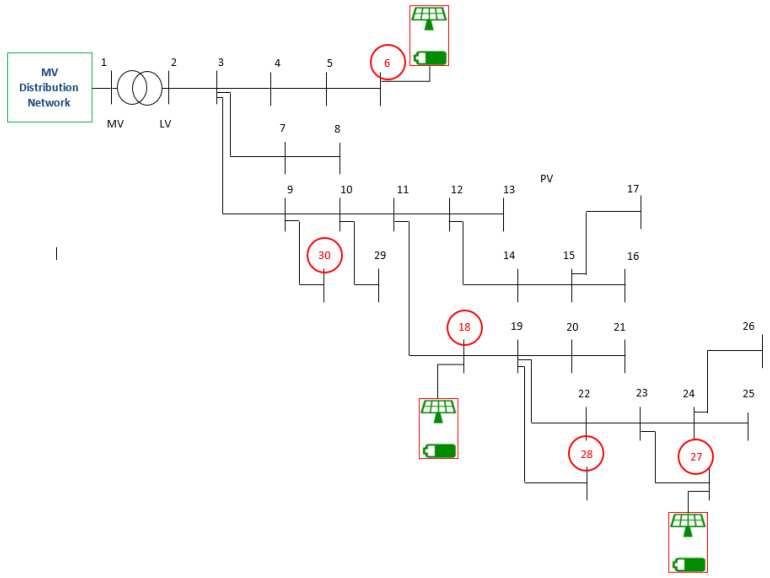
LV network considered for energy measurements.

As an example of the measurement of the energy exchanges, the Low Voltage (LV) distribution network shown in Figure 6 has been simulated through Matlab 2023a software. The LV network includes 30 buses and is connected to the upstream network through a 250 kVA transformer [29]. The REC under study includes five members connected to buses 6, 18, 27, 28, and 30, highlighted by red circles in the figure. Three of the members (i.e., those connected at buses 6, 18, and 27) include a photovoltaic (PV) generation system, each coupled with a battery energy storage system (BESS). The power exchanges between the REC members and the network are evaluated according to the optimization procedure discussed in Section 2, which is aimed at maximizing the shared energy.

**Figure 7:**
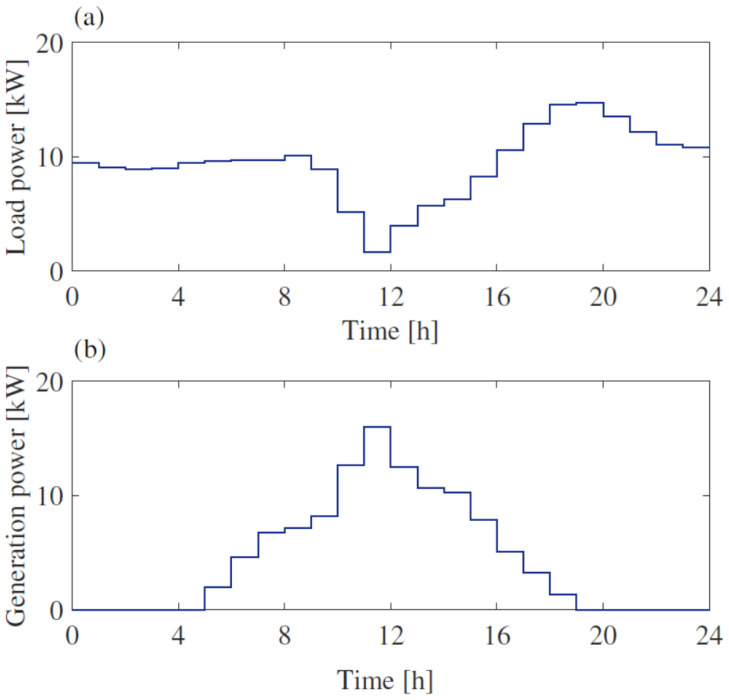
Modeled power profile for bus 6: (**a**) consumption unit (**b**) production system.

Both the PV systems connected to buses 6 and 18 have a rated power of 20 kW; the SUs have both rated capacities of 100 kWh, each with a rated power of 20 kW. Regarding bus 27, the rated power of the PV system is 50 kW, combined with an SU of 50 kW/250 kWh. Regarding the daily power profiles of loads and PV systems, the profiles related to bus 6 are shown in Figure 7. The figure refers to the hourly mean value measurements carried out during the whole day. Both generation and load daily profiles are taken in absolute values, as the measurements carried out by the unidirectional measuring devices always provide positive values.

**Figure 8:**
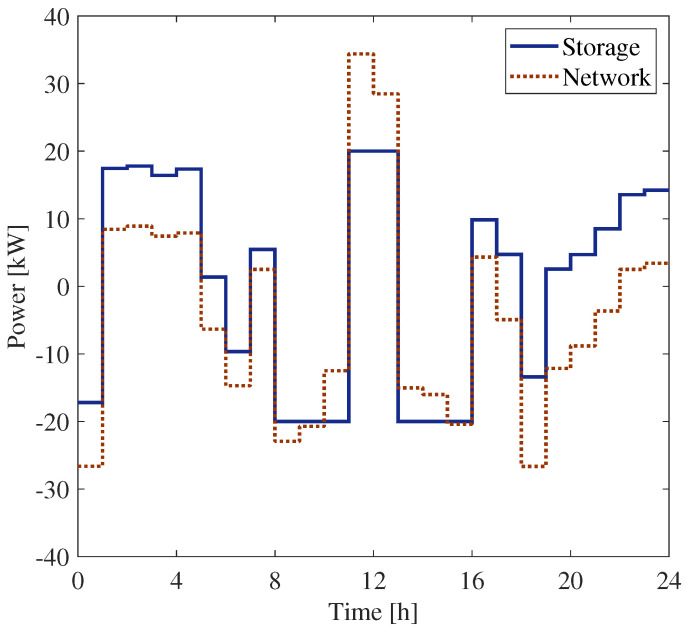
Power exchange with the network and the SU at the bus 6.

**Figure 9:**
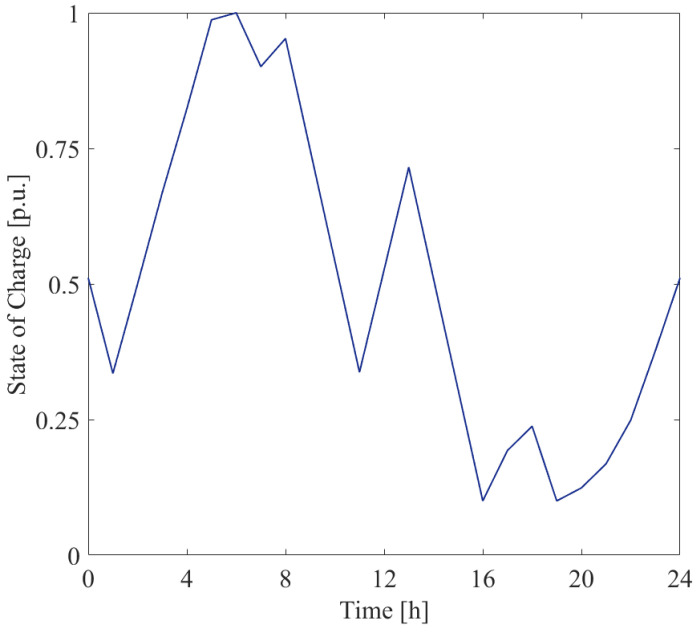
State of the charge of the SU connected at bus 6.

In Figure 8, the power measured by meters PM1 and PM3 installed at bus 6 are shown. They refer to the measurements of the BESS power and that of the overall power exchanged with the grid at bus 6. For the sake of completeness, the corresponding state of the charge at bus 6 is shown in Figure 9.

**Figure 10:**
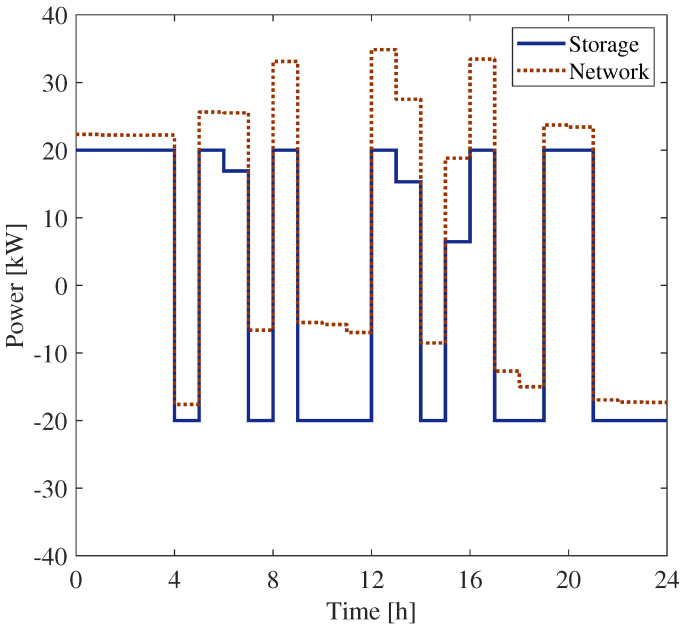
Power exchange with the network and the SU at bus 18.

**Figure 11:**
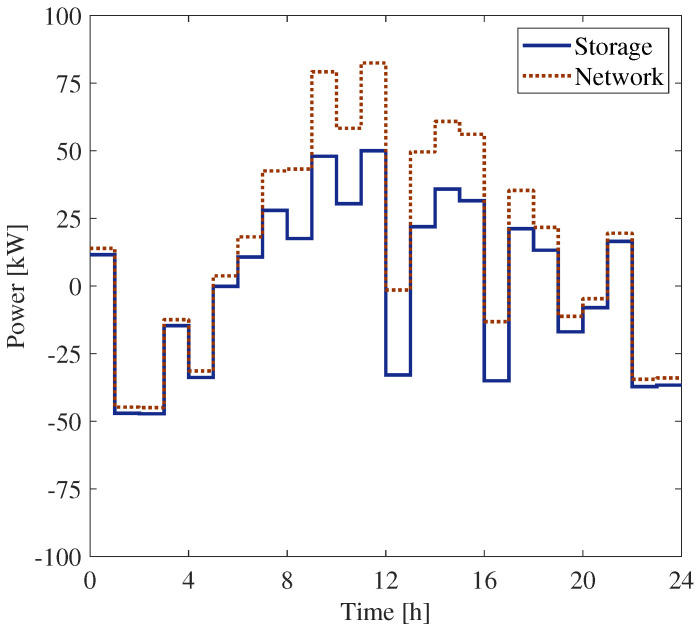
Power exchange with the network and the SU at bus 27.

Figure 10 and Figure 11 report the powers measured by meters PM1 and PM3, installed at buses 18 and 27, respectively.

**Figure 12:**
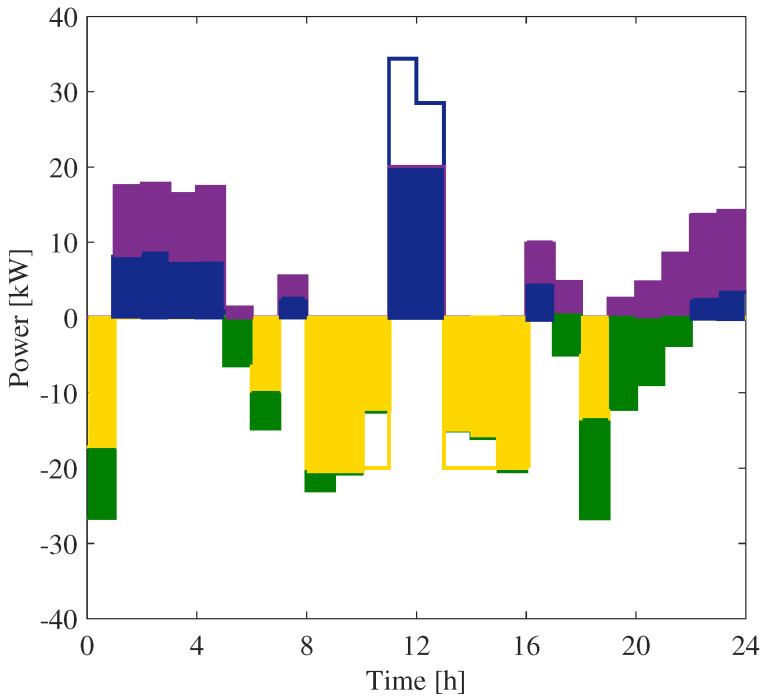
Energy contributions for bus 6.

It has to be noted that the storage power profile in the figures refers to the whole power exchanged between the SU and the grid. Thus, it also includes the power that does not contribute to the evaluation of the shared energy. According to Equation (12), the term contributing to the shared energy can be evaluated by considering the energy exchanged with the network and the production of the PS, which in the case of bus 6 are shown in Figure 7b and Figure 8, respectively. In Figure 12, the various contributions of the BESS energy exchanges are represented with different colors.

More specifically, the contributions highlighted in Figure 12 can be summarized as follows:

- The blue area represents the energy released from the SU and injected into the grid.
- The purple area is the energy released from the SU and used to feed the CU (i.e., it is not fed back into the grid).
- The yellow area represents the energy withdrawn by the SU from the grid.
- The green area is the withdrawal from the grid to feed the CU.
- The uncolored areas below zero represent the energy charged by the SU from the PS.
- The uncolored area above zero represents the energy injected by the PS into the grid.

This graph allows all users to optimize the use of community energy. For example, all users can see that, at 11:30, all the energy taken from the SU is fed into the grid and further energy is injected from the PS. In that case, it would be convenient to increase self-consumption. From Figure 12, it can be seen that when we only have measurements at the exchange point and on the SU, it is not possible to determine all the energy contributions. As an example, the energy withdrawn from the network by the SU is a part of the yellow area, and this part can be estimated according to the procedure shown in Section 2.

**Figure 13:**
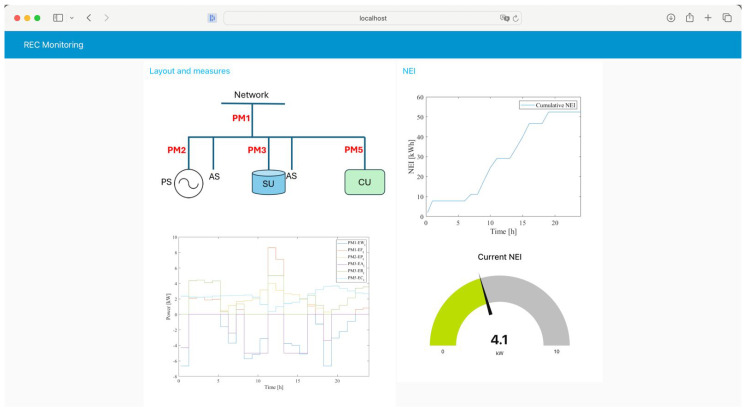
Dashboard realized in Node-RED software.

The proposed monitoring system, however, allows all energy exchanges to be determined at time *t*. As an example, Figure 13 shows the dashboard displaying the power measures provided by the power meters installed at bus 6, according to the configuration shown in Figure 2. In the displayed measurement, the NEI can also be shown in the dashboard. The partition coefficient is, in this example, evaluated assuming that the same daily power profile was repeated for all the days of the previous month. According to (11), a value equal to 0.46 is obtained for $c_{p}^{m}$. Equations (13) and (14) provide the energy charged from the SU and fed into the grid, i.e., the NEI. In the dashboard shown in Figure 13, the NEI value at time *t* and the cumulative values of the NEI, evaluated along the day, are also shown.

## 5. Conclusions

In the paper an IoT system for the monitoring of energy exchanges in a renewable energy community has been presented. The system is based on smart power meters, consisting of (1) an embedded high-accuracy power meter, able to transmit voltage, current, power, and energy measures through SPI communication; (2) a microcontroller ARM performing the power meter configuration, the data reading from the power meter, and the forwarding to the transmission unit; and (3) a transmission device able to send the measures leveraging LoRaWAN technology, and to format data according to MQTT protocol.

LoRa and MQTT were chosen for their flexibility and energy-saving characteristics. Moreover, the MQTT protocol allows energy meter data to be shared among all users in the community [30,31].

The estimated cost of a single node is about EUR 120. The largest portion of the cost is attributed to the power meter board EVALSTPM33 and the LoRaWAN transceiver. However, this is the cost of the prototype, obtained by connecting the evaluation boards. Once the device is industrialized, a board can be designed with only the necessary components, significantly reducing costs. The final cost has been estimated to be EUR 50.

The paper also analyses the placement of energy meters to ensure the accurate measurement of all energy contributions. Particular attention was given to detecting the NEI, a key parameter for incentives in energy communities. The algorithm for NEI measurement was illustrated, along with the processes for processing meter data essential for its estimation.

Finally, a dashboard was developed using Node-RED software. This tool allows community users to visualize measurements from energy meters, energy exchanges with the grid and storage system, as well as the cumulative NEI value.

The measurement algorithm and dashboard functionalities were tested through a simulation of an LV network in Matlab. The obtained results confirm that the chosen communication technology enables real-time updates of energy measurements. Additionally, the placement of the meters and the implemented algorithm ensure an accurate estimation of the energy terms.

Ongoing research focuses on the installation of the power meter in an actual REC. To this end, the study on selecting the optimal sensing technology is not yet complete. The authors are also taking into account the use of clamp-on current sensors, which would facilitate the installation of the power meters. However, the choice is not straightforward, as the cost of sensors capable of measuring both AC and DC currents with high accuracy would significantly increase the overall device cost.

Furthermore, future activities will focus on the improvement of the dashboard. This task requires collaboration with the REC members, who will help identify the most relevant measurement information and the most clear and engaging representation format.

## Data Availability

The datasets generated and/or analyzed during the study are available from the corresponding author upon request.
